# Supplementary material for: MetaQTL: a package of new computational methods for the meta-analysis of QTL mapping experiments
Source: BMC Bioinformatics. 2007 Feb 8;8:49. doi: 10.1186/1471-2105-8-49 (PMC1808479; doi:10.1186/1471-2105-8-49)
Supplement: Additional File 7 — MetaQTL Package : jar file and tutorial. This Zip archive contains both the MetaQTL JAR file and the files of the tutorial. [file 1471-2105-8-49-S7.zip › org.inra.metaqtl/doc/org/metaqtl/graph/PaletteColor.html]

PaletteColor


|  |  |  |
| --- | --- | --- |
| **Overview** \| **Package** \| **Class** \| **Use** \| **Tree** \| **Deprecated** \| **Index** \| **Help** | |  |
| **PREV CLASS**   **NEXT CLASS** | **FRAMES**    **NO FRAMES**     **All Classes** |  |
| SUMMARY: NESTED \| FIELD \| CONSTR \| METHOD | DETAIL: FIELD \| CONSTR \| METHOD |  |


---


## org.metaqtl.graph Class PaletteColor

```
java.lang.Object
  org.metaqtl.graph.PaletteColor
```

---

``` public final class PaletteColor extends java.lang.Object ```

---

| **Field Summary** | |
| --- | --- |
| `static java.awt.Color[]` | `base` |


**Constructor Summary**

`PaletteColor()`


| **Method Summary** | |
| --- | --- |
| `static java.awt.Color[]` | `getPalette(int ncolor)`             Returns a palete of colors which should be distinct. |
| `static java.awt.Color[]` | `getPaletteGradient(int nc, java.awt.Color from, java.awt.Color to)` |

**Methods inherited from class java.lang.Object**

`clone, equals, finalize, getClass, hashCode, notify, notifyAll, toString, wait, wait, wait`

**Field Detail**

### base

```
public static final java.awt.Color[] base
```


**Constructor Detail**

### PaletteColor

```
public PaletteColor()
```


**Method Detail**

### getPalette

```
public static java.awt.Color[] getPalette(int ncolor)
```

:   Returns a palete of colors which should be distinct.

    :   **Parameters:**: `ncolor` - **Returns:**

---


### getPaletteGradient

```
public static java.awt.Color[] getPaletteGradient(int nc,
                                                  java.awt.Color from,
                                                  java.awt.Color to)
```

:   **Parameters:**: `proba_bin` -: `yellow` -: `red` - **Returns:**


---


|  |  |  |
| --- | --- | --- |
| **Overview** \| **Package** \| **Class** \| **Use** \| **Tree** \| **Deprecated** \| **Index** \| **Help** | |  |
| **PREV CLASS**   **NEXT CLASS** | **FRAMES**    **NO FRAMES**     **All Classes** |  |
| SUMMARY: NESTED \| FIELD \| CONSTR \| METHOD | DETAIL: FIELD \| CONSTR \| METHOD |  |


---
